# Coarse-grained description of the spatio-temporal dynamics of network activity from experimentally verified single-neuron models and connectivity

**DOI:** 10.1186/1471-2202-16-S1-P206

**Published:** 2015-12-18

**Authors:** Francesco Fermani, Magnus JE Richardson

**Affiliations:** 1 Warwick Systems Biology Centre, University of Warwick, Coventry, UK

## 

We combine experimentally constrained models of neocortical neuron voltage dynamics [1,2] and network connectivity [3] to derive a set of equations that describe the activity at a tissue scale. The resulting equations represent a neuronal field theory in which emergent properties at a coarse-grained level can be causally linked to the physiology of cellular and sub-cellular components. The description is mathematically tractable and can be elaborated to include further biophysical details such as multiple neuronal populations to capture the structure of the component microcircuits, synaptic dynamics and filtering as well as distance-dependent delays in signal propagation. For spatially homogeneous afferent drive the steady-state firing rate can be straightforwardly calculated together with the network response to weak spatio-temporal modulation of the afferent drive via a perturbative approach. For the spatially heterogeneous non-linear regime, which the network is pushed into under stronger drive, we construct an iterative numerical scheme that rapidly converges to the network firing rate. The utility of the approach is illustrated using examples from the experimental literature.

## References

[B1] Fourcaud-Trocmé N Hansel C van Vresswijk C Brunel N How Spike Generation Mechanisms Determine the Neuronal Response to Fluctuating Inputs J Neurosci 2003 23 37 11628 14684865 10.1523/JNEUROSCI.23-37-11628.2003 PMC6740955

[B2] Badel L Lefort S Brette R Petersen CCH Gerstner W and Richardson MJE Dynamic I-V curves are reliable predictors of naturalistic pyramidal-neuron voltage traces J Neurophys 2008 99 2 656 10.1152/jn.01107.2007 18057107

[B3] Perin R Berger T K Markram H A synaptic organizing principle for cortical neuronal groups PNAS 2011 108 13 12 21383177 10.1073/pnas.1016051108 PMC3069183

